# Investigation of chromosomal aberrations in Egyptian hepatocellular carcinoma patients by fluorescence *in situ* hybridization

**DOI:** 10.4103/0971-6866.69370

**Published:** 2010

**Authors:** Magdy S. Aly, Abeer A. Bahnassy, Zekri N. Abdel-Rahman

**Affiliations:** Biological Science Department, Faculty of Science, Jazan University, Jazan, Saudi Arabia; 1 Tissue Culture Unit., Pathology Department, National Cancer Institute, Cairo University, Cairo, Egypt; 2 Virology and Immunology Unit, Cancer Biology Department, National Cancer Institute, Cairo University, Cairo, Egypt

**Keywords:** Chromosomes, genetics, hepatocellular carcinoma, interphase fluorescence *in situ* hybridization, liver cancer

## Abstract

**BACKGROUND AND AIMS:**

Hepatocellular carcinoma (HCC) is a very common and highly malignant tumor, associated mainly with chronic viral hepatitis, cirrhosis of any cause, aflatoxin exposure and ethanol consumption. Cytogenetic analysis on HCC has been limited because of poor hepatocyte growth *in vitro*. Conventional cytogenetic studies have demonstrated frequent abnormalities of specific chromosomes in HCC. Molecular cytogenetic approaches have been applied only rarely in the characterization of HCC. The main aim of this study was to evaluate genetic aberrations of different chromosomes in HCC. The study included 35 patients with HCC, who have been diagnosed and treated at National Cancer Institute, Cairo University, Egypt. The clinico-pathologic features of the studied patient were collected from patient’s files.

**MATERIALS AND METHODS:**

Interphase cytogenetics by fluorescence *in situ* hybridization with the use of a panel of centromere-associated DNA probes for chromosomes 1, 4, 8, 9, 13, 17, 20 and Y were performed on paraffin-embedded HCC specimens.

**RESULTS:**

The most common chromosomal aberrations detected were gain of chromosomes 8 in 12 cases (34.28%), 17 in 6 cases (17.14%). Loss of chromosome Y was detected in 6 of male cases (30%). Monosomy 4 was also detected in 5 cases (14.28%). Negative correlation could be detected only between chromosome 4 and 8. (r = -0.381, *P* < 0.05). Correlations between gain or loss of chromosomes and the different clinicopathologic parameters in the patients investigated, indicated negative correlation between: chromosome Y and age and chromosome 1 and cirrhosis.

**CONCLUSION:**

Gains and losses of DNA found in this study probably involve oncogenes and tumor suppressor genes that play a role in the puzzle of hepatocarcinogenesis.

## Introduction

Hepatocellular carcinoma (HCC) is among the most common malignancies worldwide. At present, approximately 550,000 new patients are diagnosed with HCC each year worldwide. However, regional differences in the incidence of HCC are significant. The highest prevalence is found in southeast Asia and the sub-Saharan Africa, mostly due to the high rates of chronic viral hepatitis, a high risk factor for HCC. Additional causes leading to HCC are alcohol, toxins such as aflatoxin, hemochromatosis, α1-antitrypsin deficiency, and non-alcoholic fatty liver disease (NAFLD).[1–5]

Yet, little is known about the molecular pathogenesis of HCC. In fact, the majority of HCC are associated with a background of chronic liver disease. Therefore, hepatocarcinogenesis is believed to be a long-term process that involves multiple genetic alterations.

Chromosome aberrations are a hallmark of solid tumors and it has been known for decades that chromosome rearrangements exist in most, if not all, human tumors. Additionally, cytogenetic study followed by molecular analysis of recurring chromosome changes has greatly facilitated the identification of crucial oncogenes and tumor suppressors.

Cytogenetic studies such as comparative genomic hybridization (CGH) and fluorescence *in situ* hybridization (FISH) have demonstrated characteristic chromosomal aberrations in conventional HCCs.[6–15] The earliest changes are gains at chromosomal arms 1q and 8q.[14,15] Other common abnormalities that occur during tumor progression are gains at 6q, 7q, 20q and X, and losses at 4q, 8p, 13q, 16q and 17p.[6–15] Some of these chromosomal changes show distinct clinicopathologic associations. Elevated alpha-fetoprotein levels and p53 mutations correlate with loss of 4q.[6] Gains of 8q and 20q have been observed in large tumors. HCCs arising in noncirrhotic liver often show gain of 8q and loss of 13q. Losses of 3q, 9p and 6q may be independent predictors of unfavorable outcome.

Frequent non-random chromosomal gains and losses detected by CGH are gains of 1q, 6p, 8q, 17q, and 20q, and losses of 1p, 4q, 5q, 6q, 8p, 9p, 10q, 13q, 16q, 17p, 19p, and 22q. In addition, the loss of heterozygosity (LOH) assay is used to define chromosomal regions with allelic deletions, and results revealed that LOH was frequently detected in 1p, 4q, 6q, 8p, 13q, 16q, and 17p.[14–16] These studies suggest the presence of multiple oncogenes or tumor suppressor genes in regions of recurrent gain or loss, respectively.

FISH is the technique that can be used to detect genetic alterations in either metaphase or interphase nuclei by appropriate probes. Interphase FISH is especially suitable for the analysis of tumor samples that are difficult to culture or that contain significant normal background cells, because it requires only intact nuclei and is evaluated on a single-cell level. So far, only few reports of interphase FISH study on HCC have been found in the literature and none of them had selected chromosomes 4, 9 as their target regions. These centromere probes are useful for aneuploid study.[16–22]

However, data on correlation of these chromosomal aberrations with the clinical course of the disease are not available, mostly due to the limited overall number of the comparatively large chromosomal aberrations and to the especially low occurrence of the same aberration within the same collective patients.

The main aim of this study was to evaluate the copy number changes in Egyptian patients with HCC. We applied FISH with (peri-) centromeric DNA probes specific for chromosomes 1, 4, 8, 9, 13, 17, 20 and Y to 35 liver tumor samples from Egyptian patients. The results were examined in relation to clinicopathologic findings to elucidate the numerical chromosomal aberrations implicated in tumor progression.

## Materials and Methods

### Patients and samples

The study included 35 patients with HCC, who have been diagnosed and treated at National Cancer Institute, Cairo University, Egypt. No preoperative radiation therapy or chemotherapy was administered to any of the patients.

Five micron thick, formalin-fixed, paraffin-embedded sections of the tumor were examined in all 35 cases. Sections were deparaffinized in xylene twice for 10 minutes, dehydrated with 100% ethanol.

### *In situ* hybridization

FISH experiments were carried out with centromeric probes for chromosomes 1, 4, 8, 9, 13, 17, 20 and Y in all 35 patients (Abbott, Wiesbaden, Germany). Pepsin digestion (99 ml of distilled water, 1 ml of 1 M HCl, and 5 mg of pepsin) for 3 minutes at room temperature was followed by washing for 1 minute in distilled water and incubation for 10 minutes in paraformaldehyde (1.5%). After washing for 1 minute in distilled water and drying the slides, 0.5 μl of each chromosome in 10 μl of hybridization buffer (Abbott) was pipetted onto the slide, placed under a glass coverslip, sealed with rubber cement, heated to 80°C for 10 minutes, and incubated overnight at 37°C in a humidified chamber. The coverslip was removed, and the slides were washed twice in 0.4× SSC and 0.3% Tween 20 at 75°C for 2 minutes. Counterstaining was done with 5 μl of 4’, 6-diamidino-2-phenylindole (40 ng/ml (Qiagen, Heidelberg, Germany). Evaluation of signals was carried out by using an epifluorescence microscope (Nikon) equipped with specific filters and a 100-W mercury lamp. To determine the cut-off levels for the detection of numerical chromosomal aberrations by using centromere-specific probes for all the chromosomes used, 2000 peripheral blood lymphocytes (i.e., 400 cells each from five healthy donors with normal karyotypes) and 2000 normal hepatocytes from liver cell aspirates of five patients with regenerative nodules and/or fatty changes were analyzed. According to Ward *et al*,[23] the thresholds for gains and losses of the respective chromosomes were calculated as the mean ± 3SD.

Statistical analysis was done using Fisher’s exact test, and the Mantel-Haenszel rank test for trend.

## Results

The study included 35 patients with HCC, who have been diagnosed and treated at National Cancer Institute, Cairo University, Egypt. The charts of the patients were reviewed to retrieve their clinicopathologic data. They were 20 males and 15 females with a male:female ratio of 1:3. Their ages ranged between 33 and 80 years (median 55 years).

The major characteristics and clinicopathologic data of the patients are summarized in Table 1.

**Table 1:** Clinical features of the studied patients

Features	Number (%)
Number of patients	
Age (years)	35 (100%)
Mean ± SD	55.14 ± 11.08
Median	55
Range	33–80
Percent of tumor cells (%)	
Mean ± SD	57.71 ± 18.29
Median	63
Range	20–90
Gender	
M	20 (57.14%)
F	15 (42.85%)
Total	35 (100%)
Cirrhosis	
Present	17 (48.57%)
Absent	18 (51.42%)
Total	35 (100%)
HCV	
Present	28 (80%)
Absent	7 (20%)
Total	35 (100%)
Grade	
I	4 (11.42%)
II	21 (60%)
III	10 (28.57%)
Total	35 (100%)
CAH	
Present	13 (37.14%)
Absent	22 (62.85%)
Total	35 (100%)

### Determination of cut-off levels

Analysis of 2000 cells from peripheral blood lymphocytes of healthy donors, with centromere-specific probes for chromosomes 1, 4, 8, 9, 13, 17 and 20 showed one signal in 2.25–3.10% of the cells (SD 1.08–1.74%) and three or more signals in 0.35 and 1.20% of cells (SD 0.29–0.78%). Thus, the cut-off levels (mean ± 3SD) were determined as 6.15–7.48% for losses and 1.21–3.14% for gains.

Analysis of 2000 normal hepatocytes from five liver aspirates with the probes mentioned above showed one signal in 2.40-3.20% of the cells (SD 0.63–1.55%) and three or more signals in 1.3–1.65% of the cells (SD 0.29–0.57%). The cut-off levels (mean ± 3SD) were determined as 4.28–7.84% for losses and 2.41-3.26% for gains. The percentage of tetrasomic cells was <1.75%.

**Figure 1:**
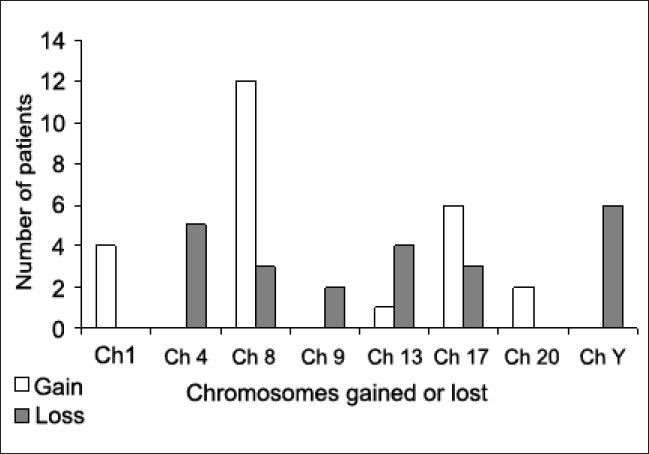
Number of patients with gain or loss of the different chromosomes

FISH was successful in all the cases studied. Most of the probes displayed a diploid spot distribution. Table 2 and Figure 1 summarize the FISH results for each of the 35 patients with numerical chromosomal aberrations. The most common chromosomal aberrations detected were gain of chromosomes 1 in 4 cases (11.42%), 8 in 12 cases (34.28%), 17 in 6 cases (17.14%). Loss of chromosome Y was detected in six of the male cases (30%). Monosomies of chromosomes 4, 8, 9, 13, and 17 were also detected in 5 (14.28%), 3 (8.57%), 2 (5.71%), 4 (11.42%) and 3 cases (8.57%), respectively.

**Table 2:** Number and percentage of patients with gain or loss of the different chromosomes

	Chromosome number							
	1	4	8	9	13	17	20	Y
Gain	4 (11.42)		12 (34.28)		1 (2.85)	6 (17.14)	2 (5.71)	
Loss		5 (14.28)	3 (8.57)	2 (5.71)	4 (11.42)	3 (8.57)		6 (17.14)

Figures in parentheses are in percentage

Table 3 shows the intercorrelations between gain and/or loss of chromosomes in the patients investigated. Negative correlation could be detected only between chromosomes 4 and 8 (*r* = –0.381, *P* < 0.05).

**Table 3:** Intercorrelations between gain or loss of chromosomes in the patients investigated

		Ch 1	Ch 4	Ch 8	Ch 9	Ch 13	Ch 17	Ch 20	Ch Y
Ch 1	R	1	0.146	–0.02	0.088	0.087	–0.238	–0.088	0.163
	P		NS	NS	NS	NS	NS	NS	NS
Ch 4	R		1	**–0.381**	–0.100	–0.099	–0.258	–0.251	0.247
	P			**<0.05**	NS	NS	NS	NS	NS
Ch 8	R			1	–0.097	0.328	–0.251	0.318	–0.315
	P				NS	NS	NS	NS	NS
Ch 9	R				1	–0.060	0.047	0.060	0.214
	P					NS	NS	NS	NS
Ch 13	R					1	0.047	0.060	–0.111
	P						NS	NS	NS
Ch 17	R						1	0.207	0.230
	P							NS	NS
Ch 20	R							1	0.111
	P								NS

Bold values are significant; NS = nonsignificant

### Clinicopathologic correlation

Table 4 demonstrates the correlations between gain or loss of chromosomes and the different clinicopathologic parameters in the patients investigated. Negative correlation could be detected between chromosome Y and age, as well as between chromosome 1 and cirrhosis.

**Table 4:** Correlations between gain or loss of chromosomes and the different clinicopathologic parameters in the patients investigated

		Ch 1	Ch 4	Ch 8	Ch 9	Ch 13	Ch 17	Ch 20	Ch Y
Age	R	–0.271	–0.105	0.241	–0.03	–0.139	–0.199	–0.091	–0.410
	P	NS	NS	NS	NS	NS	NS	NS	<0.05
% of tumor	R	–0.054	–0.366	0.325	0.003	–0.098	0.113	0.167	0.245
cells	P	NS	NS	NS	NS	NS	NS	NS	NS
Cirrhosis	R	**–0.349**	–0.256	–0.022	–0.007	0.065	0.284	0.007	–0.013
	P	**<0.05**	NS	NS	NS	NS	NS	NS	NS
HCV	R	–0.044	–0.204	–0.040	0.184	–0.122	–0.346	0.123	–0.037
	P	NS	NS	NS	NS	NS	NS	NS	NS
CAH	R	0.095	–0.024	0.2497	0.189	0.024	–0.126	0.320	–0.121
	P	NS	NS	NS	NS	NS	NS	NS
Grade	R	0.066	–0.004	0.134	–0.132	0.083	–0.151	0.132	–0.245
	P	NS	NS	NS	NS	NS	NS	NS	NS

Bold values are significant, NS = nonsignificant,

None of the other clinicopathologic parameters including sex, percentage of tumor cells, Hepatitis C Virus (HCV), Chronic Active Hepatitis (CAH) and grade, had a statistically significant correlation with the presence or absence of any of the numerical chromosomal aberrations observed in this study.

## Discussion

Knowledge about cytogenetic alterations in HCC has increased over the past few years due to the application of new techniques such as CGH and FISH. Larger numbers of HCC have now been analyzed, and recurrent patterns of chromosomal imbalances have been identified.[24–26] In particular, imbalances of chromosomes 1, 4, 6, 7, 8, and X, including total and partial gains and losses, have been demonstrated. Although not all of these aberrations were detectable in every case analyzed, at least some of them were found in varying combinations in all the HCC cases described. In this study, we used FISH as an alternative method to CGH to analyze HCC. The main reason for this approach was that FISH is easier to perform and much easier to evaluate than CGH is. Whereas CGH requires a karyotype analysis similar to conventional cytogenetics, FISH requires only the counting of single signal spots in the nuclei. Therefore, the correct identification of chromosomes, which requires a lot of experience, is not mandatory in FISH.

Centromeric probes most often give brighter signals than probes localized on the arms of the chromosomes. Evaluation of the signals can be done by epifluorescence microscopy with a standard filter set, without the need for sophisticated technical equipment.

The aneuploidy found by the panel of probes is seen not only in HCC but also in a variety of other malignant tumors affecting the same chromosomes in similar patterns, as summarized by Mitelman *et al*.[27] Lengauer *et al*.[28] discussed these findings as an increased genetic instability, based on the inability of the aberrant cell to control chromosomal alterations. This assumption is underscored by the observation that the chromosome changes found in distinct carcinomas are not always identical for all chromosomes. The mechanisms responsible for this genetic instability are not yet known and require further investigation.

Molecular genetic analyses have identified the loss of heterozygosity at many loci in HCCs with the high frequencies at 1p, 4q, 11p, 13q, 16q and 17p.[29–34]

The basic technique for the detection of chromosomal imbalances is the classic cytogenetic examination (CG). For CG, tumor cells are cultivated *in vitro* with subsequent preparation of metaphases or chromosomes. Cell culture results in selective cell growth and secondary changes in chromosomal material. CG is difficult to perform in solid tumors such as HCC. Until now, about 20 primary HCC or HCC cell lines have been investigated cytogenetically. Recurrent aberrations of chromosomes 1q, 4q, 6q, 8p, 8q, 16p and 17 have been found.[35–38] Due to these limitations, CG cannot be recommended for diagnostic purposes.

The purpose of our study was to characterize numerical aberrations of certain chromosomes during hepatocarcinogenesis. This study was useful in identifying sequential genetic events associated with the progression of HCC. However, characterization of the cytogenetic pathway to hepatocarcinogenesis will require examination of both borderline lesions and small carcinomas because the earliest genetic events responsible for disease development have likely been overshadowed by the numerous genetic abnormalities present in advanced carcinomas.

In our study, we used a panel of centromere-associated DNA probes for chromosomes 1, 4, 8, 9, 13, 17, 20 and Y on paraffin-embedded HCC specimens from Egyptian patients.

So far, only a few reports of interphase FISH study on HCC have been found in the literature.[16–22] Huang *et al*.,[22] using centromeric probes for chromosomes 3, 4, 6, 8 and 9, showed at least one deletion or aneuploidy for chromosomes 4 and 8. Hamon-Benais *et al*.[18] demonstrated numerical changes in chromosomes 7, 17 and 20 in all six HCCs examined. Trisomy 1 and 8 has been frequently encountered in HCCs.[16] The numerical abnormalities of chromosome 17 were associated with increased histologic grade and proliferative activity.[17] Numerical chromosome aberrations occurred in HCC from early-stage patients and became more prominent with severe histologic grades and tumor progression.[39]

In our series, the most common chromosomal aberrations detected were gain of chromosomes 8 in 12 cases (34.28%) and 17 in 6 cases (17.14%). Loss of chromosome Y was detected in six of the male cases (30%). Monosomy 4 was also detected in five cases (14.28%). Negative correlation could be detected only between chromosomes 4 and 8. (*r* = –0.381, *P* < 0.05). Our results in Egyptian patients are in agreement with those of other reports. Huang *et al*.[40] described frequent allelic loss at 4q21, 8p22, and 6q14 by FISH, using yeast artificial chromosome (YAC) probes in 17 cases of HCC. Frequent deletion on 4q and 8p in HCC has been reported by various studies using microsatellite polymorphism, and in one study using comparative genomic hybridization.[41–45] This indicated that loss or inactivation of tumor suppressor genes in these loci may play a major role in the development of HCC. Other chromosomal sites that have been reported to be deleted in HCC include 1p, 5q, 6q,10q, 11p, 16q, 17p and 22q.[43–45]

In conclusion, we have demonstrated the utility of FISH technique in evaluating HCC clonal cytogenetic aberrations. Implantation of extended panels of FISH probes will provide a mechanism complementary to allelic imbalance (loss of heterozygosity) analysis for the characterization of specific regions. Gains and losses of DNA found in this study probably involve oncogenes and tumor suppressor genes that play a role in the puzzle of hepatocarcinogenesis.
